# Enhancement of hippocampal synaptic AMPA receptor function by the antidepressant tianeptine and by opioid peptides

**DOI:** 10.1073/pnas.2521987123

**Published:** 2026-10-07

**Authors:** Anna Mariano, Andrei Rozov, Grant D. Phillips, Sam Singleton, Daniel Baptista-Hon, Fliza Valiullina-Rakhmatullina, Rolf Sprengel, Rosamund Langston, Stephen J. Martin, Tim G. Hales, Jeremy J. Lambert

**Affiliations:** ^a^ Division of Neuroscience, School of Medicine, University of Dundee, Dundee DD1 5HL, United Kingdom; ^b^ Institute of Polymers, Composites and Biomaterials—Consiglio Nazionale delle Ricerche, Naples 80125, Italy; ^c^Federal Center of Brain Research and Neurotechnologies, Moscow 117513, Russia; ^d^ Laboratory of Neurobiology, Institute of Fundamental Medicine and Biology, Kazan Federal University, Kazan 420008, Russia; ^e^Faculty of Medicine, Macau University of Science and Technology, Macau SAR 999078, China; ^f^Macau Institute for AI in Medicine, Macau University of Science and Technology, Macau SAR 999078, China; ^g^ Max Planck Institute for Medical Research, Heidelberg D-69120, Germany

**Keywords:** tianeptine, AMPA receptors, opioid receptors, antidepressants

## Abstract

Management of major depressive disorder (MDD) with conventional therapeutics that enhance monoaminergic neurotransmission requires prolonged treatment prior to clinical benefit and a substantial proportion of patients show little or no improvement. Identifying alternative molecular targets is required to develop efficacious, rapid acting antidepressants. In this regard, tianeptine, an established but atypical clinical antidepressant, is of interest. Tianeptine does not directly influence monoamine levels; in rodents it has a relatively rapid onset of antidepressant-like efficacy and, unusually for an antidepressant, exhibits procognitive properties. Here, voltage-clamp recordings from mouse hippocampal CA1 pyramidal neurons demonstrated tianeptine enhanced the amplitude of neurally evoked excitatory postsynaptic currents mediated by glutamate activation of synaptic α-amino-3-hydroxy-5-methyl-4-isoxazolepropionic acid receptors (AMPARs). The effect required intracellular polyamines and expression of the GluA1 subunit. These observations suggest that tianeptine relieves the channel block by endogenous polyamines of synaptic GluA1 containing AMPARs (GluA1-AMPARs). The tianeptine effect was abolished by postsynaptic inhibition of G-protein signaling, was reduced in mice lacking either the µ- or the δ-opioid receptor, and inhibited by selective antagonists of these receptors, collectively demonstrating that the facilitation of excitatory transmission was indirectly mediated by activation of opioid receptors. Importantly, enhancement of GluA1-AMPAR function was mimicked by opioid peptide and nonpeptide agonists selective for µ- or δ-receptors. These observations improve understanding of how the antidepressant tianeptine enhances glutamatergic transmission but more generally suggest that endogenous and exogenous opioids act in a similar manner, which may contribute to their behavioral effects.

## Significance

There is a need to identify targets to develop efficacious treatments for depressive disorders. Tianeptine is a clinical antidepressant of unknown mechanism that unusually exhibits procognitive properties. Here, tianeptine enhanced hippocampal glutamatergic transmission by reducing the resident block of Ca^2+^-permeable α-amino-3-hydroxy-5-methyl-4-isoxazolepropionic acid receptors (CP-AMPARs) by intracellular polyamines. Intriguingly, this effect is indirectly mediated by activation of opioid receptors and is mimicked by opioid agonists. In common, drugs that facilitate AMPAR function and opioid receptor agonists exhibit antidepressant-like effects in rodents. This form of synaptic receptor modulation may contribute to the behavioral effects of tianeptine, but more generally provides a mechanism whereby both exogenous synthetic and endogenous opioids may influence mood and cognition.

Depressive disorders are prevalent psychiatric conditions, estimated by the World Health Organization to affect ~350 million individuals worldwide (1). Most current antidepressants influence the levels of biogenic amines such as serotonin and norepinephrine. However, such treatments require weeks to take effect and a substantial proportion of patients experience only limited or no improvement (2, 3). Identification of alternative targets is required to aid development of effective, rapidly acting antidepressants (4). In this context the clinical atypical antidepressant tianeptine is of interest. Tianeptine does not bind to transporters and enzymes for monoamines, or to receptors for serotonin, norepinephrine, and dopamine (3–5). A clinical study reported therapeutic efficacy within a week, with a similar timeline for the emergence of antidepressant-like actions in mice (6, 7). Further distinguishing tianeptine, in rodents tianeptine not only displays antidepressant-like activity in stress-associated paradigms, but unusually exhibits procognitive efficacy, a clinically useful attribute given the prevalence of memory dysfunction in patients suffering from MDDs (8, 9).

Some studies have focused on an effect of tianeptine that is not directly due to monoamines but to facilitation of fast glutamatergic transmission and synaptic plasticity mediated by α-amino-3-hydroxy-5-methyl-4-isoxazolepropionic acid receptors (AMPARs) (5, 10–12). Clinical and preclinical evidence implicates perturbations of glutamatergic transmission and synaptic plasticity in major depressive disorders (13–16). Furthermore, compounds that enhance AMPAR function exhibit antidepressant-like properties in rodent models of depression and efficacy in preliminary clinical trials (16–19). We and others demonstrated that tianeptine acutely increased hippocampal field excitatory postsynaptic potentials (fEPSPs) mediated by synaptic AMPARs and facilitated long term potentiation (LTP), a form of synaptic plasticity associated with memory processes (11, 12). Clinically, chronic stress is a risk factor associated with depression (15, 20, 21). In rodents tianeptine rescued the impaired LTP resultant from either acute stress or caused by corticosterone (10, 12, 22).

AMPARs are tetrameric protein assemblies drawn from a repertoire of GluA1-4 subunits (23, 24). In hippocampal neurons tianeptine reduced the cell surface diffusion of AMPARs incorporating GluA1 and GluA2 subunits, favoring their synaptic retention and countered perturbations of AMPAR subunit trafficking by corticosterone (12). Further implicating AMPARs, tianeptine increased phosphorylation of key serine residues (S831, a CaMKII site and S845, a PKA site) of the GluA1 subunit, known posttranslational modifications that influence AMPAR function (5, 11). In mice GluA1(S831) of AMPARs is an important locus for the antidepressant-like actions of serotonin uptake inhibitors (13). Importantly, the effect of tianeptine to reduce immobility in the tail suspension test and to enhance locomotor activity was blunted in mice harboring a phosphorylation-resistant mutation (serine to alanine) of these GluA1 S831 and S845 residues (5), implicating GluA1-AMPARs in the behavioral effects of this antidepressant.

Here we employed the whole-cell voltage-clamp technique to investigate the actions of tianeptine upon synaptic AMPARs in mouse CA1 pyramidal neurons. Using wild-type (WT) mice and *Gria1^–/–^*mice lacking GluA1 subunit expression (25), we show that tianeptine enhanced glutamatergic transmission through a mechanism that selectively reduced the block of synaptic GluA1-containing AMPARs by endogenous intracellular polyamines. However, this effect is indirect, as it is prevented by blocking postsynaptic G-protein signaling with intracellular GDP-βS. Biochemical studies revealed tianeptine to activate δ- and µ-, but not κ-receptors (3). Here, the AMPAR-enhancing effect of tianeptine was reduced by genetic deletion of the δ- or the µ- receptor, was inhibited by specific δ- or µ- receptor antagonists and was mimicked by selective opioid peptide and nonpeptide agonists of these receptors.

This mechanism, whereby the activation of a G-protein-coupled receptor (GPCR) facilitates the unblocking by endogenous polyamines of a transmitter-gated ion channel, represents a distinctive form of drug-induced synaptic plasticity. As compounds that enhance glutamatergic transmission exhibit antidepressant-like effects, these findings may improve the understanding of the mechanisms that contribute to the behavioral effects of tianeptine. Furthermore, given enkephalinase inhibitors and δ-receptor agonists, including enkephalins, also exhibit antidepressant-like actions in animal studies, these observations suggest that endogenous opioid peptides may similarly influence glutamatergic transmission (26–29).

## Results

### Tianeptine Enhancement of AMPAR-Mediated Evoked Excitatory Postsynaptic Currents (eEPSCs) Is Polyamine Dependent.

**Fig. 1.**
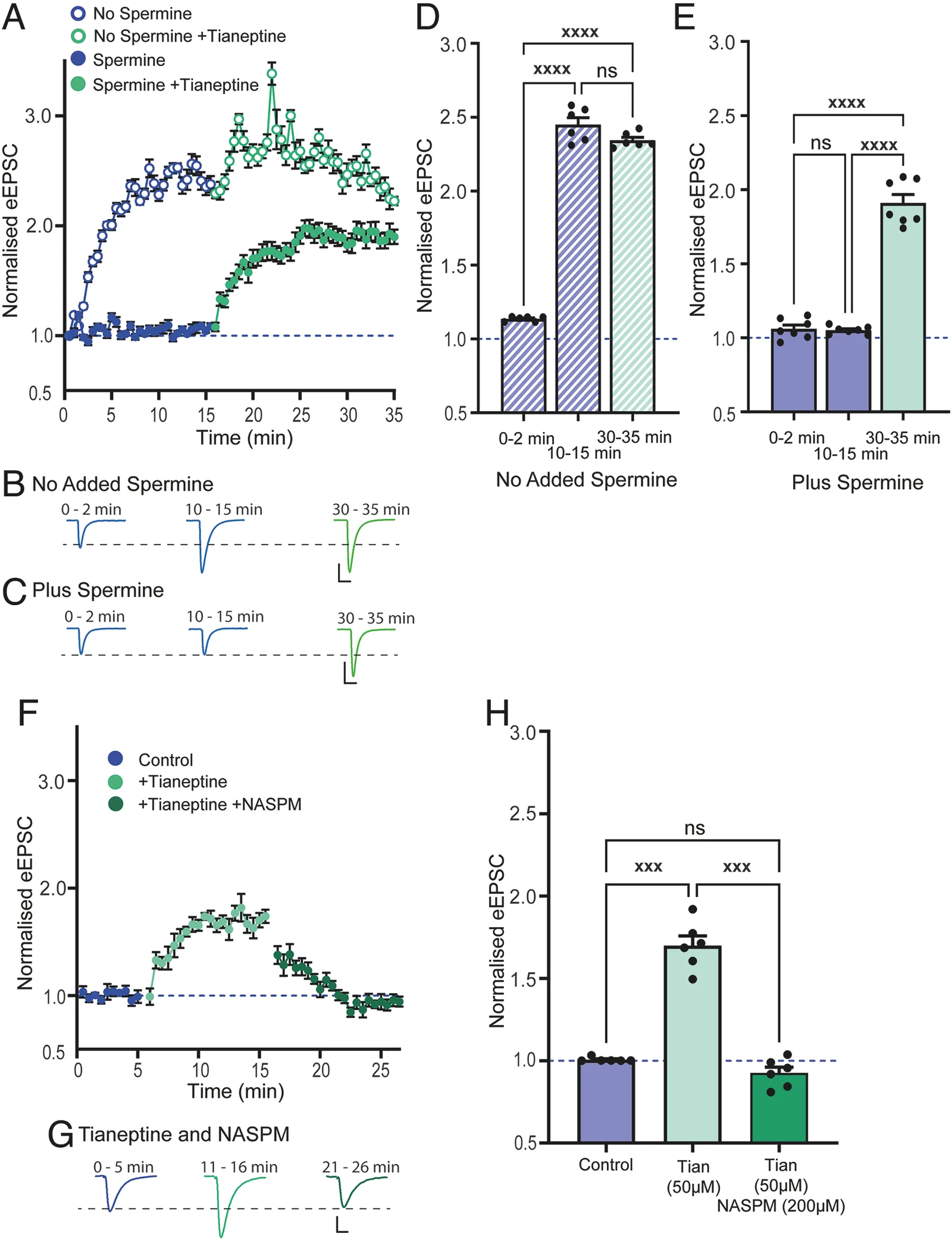
Tianeptine enhances synaptic AMPAR function by relieving the endogenous polyamine block. eEPSCs were evoked by stimulation of the Schaffer collateral pathway once every 6 s. Having established a whole-cell recording (Vh = −70 mV), the eEPSC amplitude of the first 5 events (0 to 30 s) appeared stable. The mean of these control (0 to 30 s) eEPSCs, was normalized to 1 for each neuron. (*A*) A plot illustrating the eEPSC amplitude (mean ± SEM), with time from “breaking into” the CA1 pyramidal neuron at 0 min. The recording electrode contained either a polyamine-free intracellular solution (ICS) (n = 6, 3 mice), or 100 µM spermine (n = 7, 3 mice). Without intracellular spermine, the eEPSC amplitude was stable for the first 5 events (0 to 30 s), but then increased, stabilizing at 10 to 15 min (2.45 ± 0.05). Subsequent application of tianeptine (50 µM) had no further effect on the eEPSC amplitude (30 to 35 min = 2.34 ± 0.02). By contrast, with intracellular spermine the eEPSC amplitude was stable (10 to 15 min = 1.05 ± 0.01), but now the subsequent application of tianeptine substantially increased the eEPSC amplitude assessed between 30 and 35 min (1.91 ± 0.06). (*B* and *C*) Representative averaged eEPSCs recorded at the times specified with the dotted line indicating the amplitude of the normalized eEPSC at 0 to 30 s, for (*B*) in the absence and (*C*) presence of intracellular spermine (100 µM), before (blue) and following tianeptine (green). (*D* and *E*) Histograms of the eEPSC amplitude recorded at the times indicated with the dotted line indicating the normalized eEPSC amplitude between 0 and 30 s for (*D*) no added spermine (hatched columns) for (*E*) with intracellular 100 µM spermine (filled columns), before (blue) and after (green) tianeptine. (*D*) A repeated measures one-way ANOVA revealed a significant effect of time (F(1.3, 6.7) = 750.0; *P* < 0.0001). Further analysis (Bonferroni’s multiple comparison post hoc test) identified significant differences between 0 and 2 min vs. 10 to 15 min (*P* < 0.0001) and 0 to 2 min vs. 30 to 35 min (*P* < 0.0001), but no significant difference between 10 to 15 min vs. 30 to 35 min (ns = *P* > 0.05) i.e., following “polyamine washout” tianeptine was inert. (*E*) A repeated measures one-way ANOVA revealed a significant effect of time (F(1.3, 7.8) = 202.5; *P* < 0.0001). Further analysis (Bonferroni’s multiple comparison post hoc test) found no significant difference between 0 and 2 min vs. 10 to 15 min (ns = *P* > 0.05), i.e., the eEPSC amplitude remained stable over this period. However, 0 to 2 min vs. 30 to 35 min (*P* < 0.0001), and 10 to 15 min vs. 30 to 35 min comparisons were significantly different (*P* < 0.0001), i.e., tianeptine significantly enhanced the eEPSC amplitude in the presence of intracellular spermine. (*F*) A time plot of the eEPSC amplitude (mean ± SEM) with intracellular spermine (100 µM), followed by application of tianeptine (50 µM) and the subsequent coapplication of NASPM (200 µM). (*G*) Representative averaged eEPSCs recorded at the times specified indicate the amplitude of the control eEPSC (blue), in tianeptine (light green), and in tianeptine + NASPM (dark green). (*H*) A histogram of the effect of tianeptine (50 µM) on the eEPSC amplitude and the subsequent coperfusion of 200 µM NASPM (n = 6, 4 mice). A repeated measures one-way ANOVA revealed a significant effect of treatment (F(1.6, 7.8) = 111.0; *P* < 0.0001), a Bonferroni’s multiple comparison post hoc test found a significant difference between control (1.0 ± 0.005) and tianeptine (1.7 ± 0.06; *P* < 0.001). The subsequent coperfusion of NASPM with tianeptine (0.9 ± 0.04) significantly reduced eEPSC peak amplitude compared to 50 µM tianeptine alone (*P* < 0.001) back to control (*P* > 0.05). (*D*, *E*, and *H*). For all histograms each column represents the mean ± SEM of the eEPSC amplitude. xxxx = *P* < 0.0001, xxx = *P* < 0.001), ns = nonsignificant. For (*B*, *C*, and *G*) the calibration bar for the traces is y-axis = 50 pA, x-axis = 10 ms.

Whole-cell voltage-clamp recordings, made at a holding potential (Vh) of −70 mV, were performed with mouse hippocampal CA1 pyramidal neurons. Subthreshold eEPSCs were obtained by stimulating the Schaffer collateral pathway once every 6 s. Having established a whole-cell recording, the eEPSC amplitude of the first 5 events (0 to 30 s) appeared stable before subsequently increasing. The mean of these initial control (0 to 30 s) eEPSC amplitudes was normalized to 1. During the following 10 min the eEPSC peak amplitude increased considerably, before stabilizing at a new steady state level (10 to 15 min = 2.45 ± 0.05; n = 6; Fig. 1 *A*, *B*, and *D*). When compared to the now stabilized eEPSC amplitude, the subsequent application of tianeptine (50 µM) had no further effect (30 to 35 min = 2.34 ± 0.02; n = 6; Fig. 1 *A*, *B*, and *D*). For whole-cell recordings, we demonstrated that the initial increase of synaptic AMPAR responses in CA1 pyramidal neurons results from the “washout” of endogenous intracellular polyamines e.g., spermine, which act as endogenous inhibitors of GluA2-lacking Ca^2+^-permeable (CP)-AMPAR channels (30). In agreement, with spermine (100 µM) added to the recording pipette, the control responses now remained stable (10 to 15 min = 1.05 ± 0.01; n = 7; Fig. 1 *A*, *C*, and *E*). Importantly, the subsequent application of tianeptine (50 µM) to CA1 neurons, supplemented with intracellular spermine, now produced a clear enhancement of the eEPSC amplitude (30 to 35 min = 1.91 ± 0.06, n = 7; Fig. 1 *A*, *C*, and *E*). Collectively, these observations suggest that tianeptine enhanced the function of synaptic AMPARs by relieving the endogenous polyamine block of CP-AMPAR channels. For subsequent experiments, spermine was included in the recording pipette unless otherwise stated. CP-AMPAR channels are blocked by the extracellular application of spermine analogs such as 1-naphthylacetylspermine (NASPM) (31, 32). The increased eEPSC amplitude produced by 50 µM tianeptine (1.7 ± 0.06, n = 6) was completely reversed by the subsequent coapplication of 200 µM NASPM (0.93 ± 0.04, n = 6), further implicating CP-AMPAR channels and polyamines in the AMPAR-enhancing actions of tianeptine (Fig. 1 *F*–*H*). Note polyamines, including spermine and NASPM block other cation-conducting channels, including N-methyl-D-aspartate (NMDA) receptor channels (33–35). However, under our recording conditions [Vh = −70 mV, intracellular spermine (100 µM) and an extracellular solution containing 2.5 mM MgCl_2_] the NMDA receptor antagonist AP5 (50 µM) had no significant effect on the eEPSC amplitude, suggesting that in this experimental setting NMDA receptors do not contribute to the eEPSC peak amplitude (*SI Appendix*, Fig. S1).

### For Hippocampal CA1 Pyramidal Neurons, the AMPAR-Modulatory Effect of Tianeptine Is Mediated by Postsynaptic GluA1-AMPARs.

**Fig. 2.**
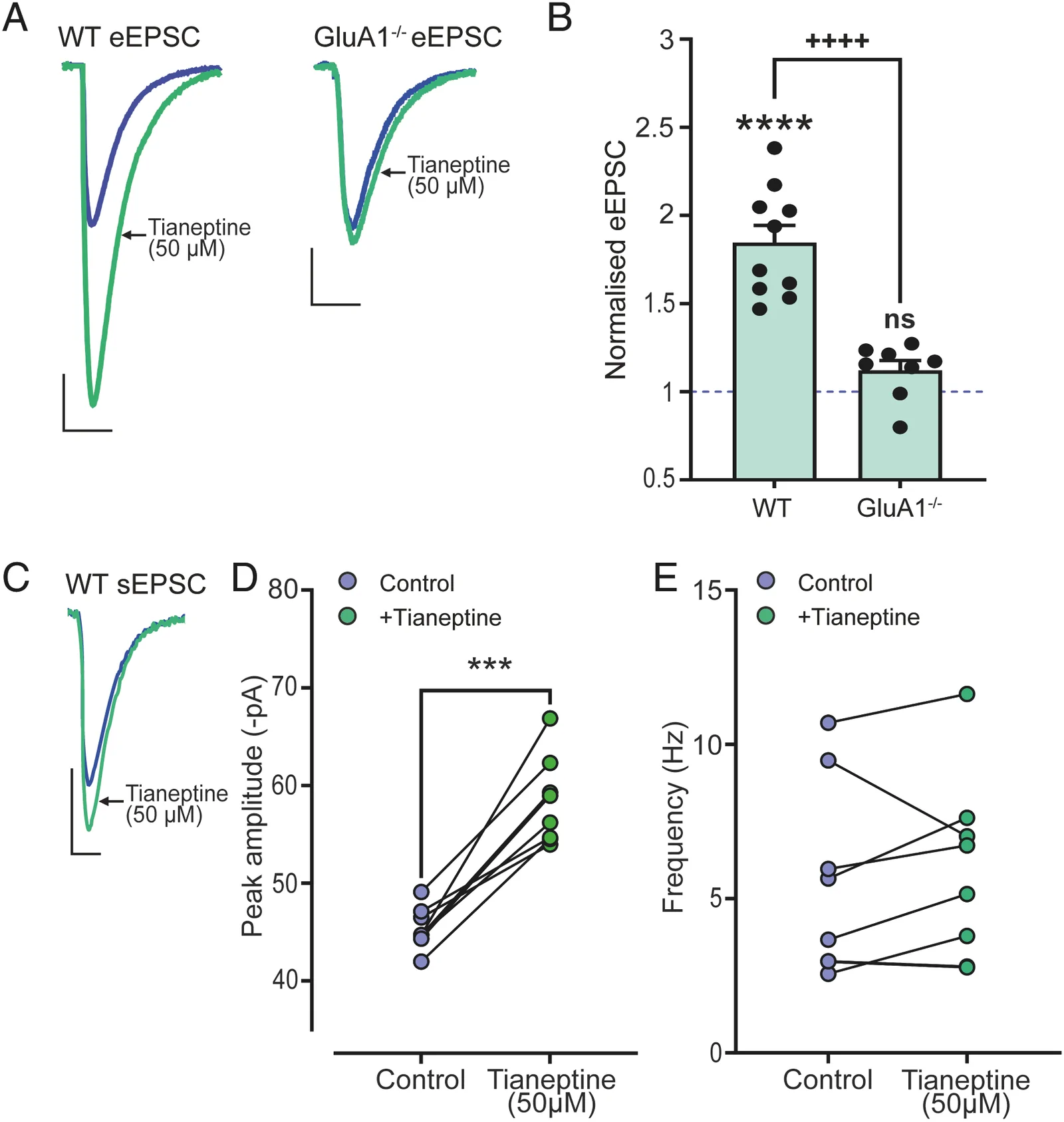
Enhancement by tianeptine of glutamatergic neurotransmission is selectively mediated by postsynaptic GluA1-AMPARs. (*A*) Representative traces illustrating an ensemble average of eEPSCs recorded from a WT and a *Gria1^–/–^* mouse CA1 pyramidal neuron in the presence of intracellular spermine (100 µM) before (blue trace) and 10 min after 50 µM tianeptine perfusion (green trace) (y-axis = 50 pA, x-axis = 10 ms). (*B*) A histogram comparing the tianeptine effect upon the normalized eEPSC peak amplitude of neurons from the two genotypes. Each column represents the influence of tianeptine on the eEPSC amplitude (mean ± SEM). Initial analysis established a significant difference in the effect of tianeptine between the two genotypes, (++++ = *P* < 0.0001, unpaired Student’s *t* test). Further investigation (one-sample *t* test) revealed that tianeptine produced a significant enhancement of the eEPSC amplitude of WT neurons (1.85 ± 0.10; n = 10, 6 mice, **** = *P* < 0.0001) but had no significant effect on the eEPSC peak amplitude of neurons obtained from *Gria1^–/–^* mice (1.12 ± 0.06; n = 8, 3 mice; ns = *P* > 0.05). (*C*) A paired continuous recording from a single WT neuron with intracellular spermine (100 µM) of averaged sEPSCs before (blue trace) and 10 min after (green trace) the perfusion of tianeptine (50 µM)–(calibration bar y-axis = 20 pA, x-axis = 5 ms). (*D* and *E*) Before and after plots illustrating the effect of tianeptine on the sEPSC (*D*) amplitude (pA) and (*E*) frequency (Hz) for individual neurons. For each neuron, the properties of >50 events were assessed. Tianeptine (*D*) significantly enhanced the sEPSC amplitude (control = −45 ± 0.8 pA; tianeptine = −58 ± 1.6 pA; n = 8, 4 mice; *** = *P* < 0.001; Student’s paired *t* test), but (*E*) had no significant effect upon their frequency of occurrence (control = 5.5 ± 1.1 Hz; tianeptine = 6.0 ± 1.0 Hz; n = 8, 4 mice; *P* > 0.05 Student’s paired *t* test). n = number of neurons.

The polyamine blocking effect of AMPARs depends upon their subunit composition, with receptors composed of GluA1, GluA3, GluA4 subunits, or a nonedited form of the GluA2 subunit, being polyamine sensitive and the dominantly expressed edited form of the GluA2 subunit being polyamine insensitive (23, 32). The increased eEPSC amplitude in the absence of supplemented polyamine (Fig. 1 *A*, *B*, and *D*), does not occur in CA1 pyramidal neurons derived from *Gria1^−/−^* mice (GluA1^−/−^) or from *Gria1^(Q600R)^* mice (GluA1R) where the polyamine sensitivity was abolished by substituting a critical glutamine(Q) for an arginine residue (R) at the “Q/R site” (25, 30, 36). We now investigated the effect of tianeptine upon CA1 GluA1^−/−^ pyramidal neurons in the presence of intracellular spermine. Tianeptine (50 µM) again produced a significant enhancement of the eEPSC amplitude of WT CA1 neurons, (1.85 ± 0.10; n = 10) but was ineffective on the eEPSC amplitude of CA1 GluA1^−/−^ neurons (1.12 ± 0.06; n = 8; Fig. 2 *A* and *B*). Collectively, these results demonstrate that tianeptine enhanced eEPSCs by influencing the resident polyamine block of synaptic GluA1-AMPARs. The spermine requirement in the CA1 neuron for the tianeptine effect upon eEPSCs suggests that it acts postsynaptically. In support, tianeptine (50 µM) had no effect upon the frequency of spontaneously occurring EPSCs (sEPSC) (control = 5.5 ± 1.1 Hz; tianeptine = 6.0 ± 1.0 Hz; n = 8 neurons) but enhanced their amplitude (control = −45 ± 0.8 pA; tianeptine = −58 ± 1.6 pA; n = 8 neurons; Fig. 2 *C*–*E*).

### Comparing the Effects of Tianeptine and LTP on Synaptic AMPAR Function.

**Fig. 3.**
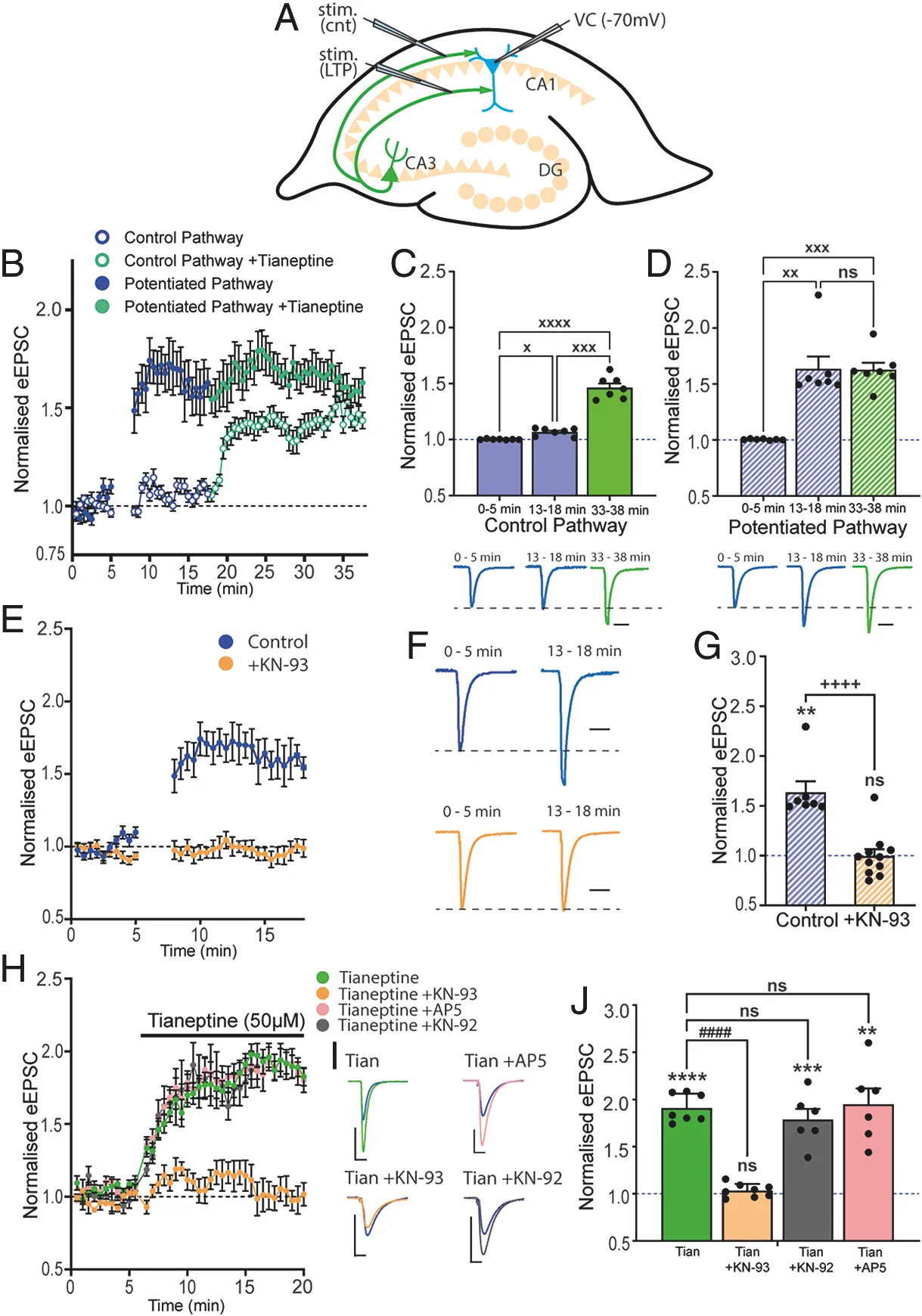
Prior LTP occludes the effect of tianeptine to enhance synaptic AMPARs. (*A*) A schematic diagram of the experimental design employed to electrically stimulate two spatially separated synaptic inputs from CA3 pyramidal cells innervating the apical and basal dendrites in the Str. radiatum and Str. Oriens respectively of a CA1 pyramidal neuron. (*B*) The time course of the effect of tianeptine (50 µM) on the eEPSC amplitude elicited by stimulation (once every 6 s, averaged into 30 s bins) of the Str. oriens control pathway and the Str. radiatum potentiated pathway. Each point represents the mean ± SEM (n = 7, 5 mice). All recordings were made in the presence of intracellular spermine (100 µM). Prior to tianeptine, at 5 min (indicated by the break in the time axis) the apical input was potentiated (LTP) by applying the pairing stimulation protocol (37, 42), consisting of coupling depolarization of the postsynaptic pyramidal cell to 0 mV with stimulation of the inputs to the apical dendrites every 1.5 s (repeated 120 times). The stimulus intensity and duration were the same for the control recordings, although it was switched off during the pairing protocol. Electrical stimulation of both pathways (1/6 s) was recommenced at 8 min. The pairing protocol resulted in LTP (1.64 ± 0.11). determined at 13 to 18 min, i.e., 5 to 10 min after recommencing stimulation. Following establishment of LTP, tianeptine had no further effect upon the eEPSC amplitude (33 to 38 min = 1.63 ± 0.06). However, in contrast tianeptine clearly enhanced the eEPSC amplitude evoked by stimulation of the control pathway (33 to 38 min = 1.46 ± 0.04). (*C* and *D*) Histograms of the eEPSC amplitude resulting from stimulation of the (*C*) control (filled columns) and (*D*) potentiated pathway (hatched columns) before (blue) and after (green) tianeptine, with representative eEPSCs provided below at the times indicated. (calibration bar = 10 ms). (*C*) For the control pathway a repeated measures one-way ANOVA revealed a significant effect of time (F(1.21, 7.28) = 127.6; *P* < 0.0001) and a Bonferroni’s multiple comparison identified a significant increase of the eEPSC amplitude by tianeptine comparing recordings at 0 to 5 min to those at 33 to 38 min (xxxx = *P* < 0.0001) and recordings at 13 to 18 min to 33 to 38 min (xxx = *P* < 0.001). (*D*) For the potentiated pathway a repeated measures one-way ANOVA revealed a significant effect of time (F(1.25, 7.51) = 38.9, *P* < 0.001) and a Bonferroni’s multiple comparison identified a significant increase of the eEPSC amplitude at both 13 to 18 min (xx = *P* < 0.01) and 33 to 38 min (xxx = *P* < 0.001) when compared to 0 to 5 min, but with no significant difference between 13 to 18 min and 33 to 38 min (ns = *P* > 0.05) i.e., no effect of tianeptine following LTP. (*E*) The time course of the effect of the pairing LTP protocol (indicated by a break in the X axis) on the eEPSC amplitude for control and with intracellular 10 µM KN-93. (*F*) Representative traces of eEPSCs for the control (blue) and the KN-93 (orange) condition, at the times indicated (calibration bar = 10 ms). (*G*) A histogram showing the magnitude of LTP in control (blue hatched column, 1.64 ± 0.11 n = 7, 5 mice) and with intracellular (10 µM) KN-93 (orange hatched column, 1.00 ± 0.07 n = 11, 5 mice). There was a significant difference between the two conditions (++++ = *P* < 0.0001, unpaired *t* test). Further analysis revealed the control eEPSC was significantly enhanced by the pairing protocol (** = *P* < 0.01. one-sample *t* test), but not with KN-93 (ns = *P* > 0.05, one-sample *t* test). (*H*) The time course of tianeptine (50 µM) on the eEPSC amplitude and for tianeptine 1) plus intracellular 10 µM KN93, 2) plus intracellular 10 µM KN92 and 3) extracellular 100 µM AP5 (pink symbols). (*I*) Representative averaged control eEPSCs (blue), superimposed with those recorded with tianeptine (green) in the presence of KN-93 (orange), KN-92 (gray), and AP5 (pink). Calibration y-axis = 50 pA, x-axis = 10 ms. (*J*) A histogram illustrating the effect of tianeptine on the eEPSC amplitude in control (green column 1.9 ± 0.06 n = 7, 3 mice), with intracellular (10 µM) KN-93 (1.0 ± 0.03 n = 8, 7 mice), with intracellular (10 µM) KN-92 (gray column 1.8 ± 0.11, n = 6, 3 mice) and extracellular (100 µM) AP5 (pink column 1.95 ± 0.17 n = 6, 6 mice). There was a significant effect of treatment (F(3, 23) = 22.85, one-way ANOVA). In comparison to tianeptine alone a Dunnett’s multiple comparison post hoc analysis revealed the effect of tianeptine to be significantly reduced in the presence of intracellular KN-93 (#### = *P* < 0.0001) but not KN-92 (ns = *P* > 0.05) or AP5 (ns = *P* > 0.05). Subsequent one-sample *t* tests demonstrated that tianeptine (50 µM), produced a significant increase of the eEPSC amplitude in the absence (**** = *P* < 0.0001) and presence of KN-92 (*** = *P* < 0.001) and AP5 (** = *P* < 0.01), but had no significant effect in the presence of intracellular KN-93 (ns = *P* > 0.05). For all histograms, each column represents the mean ± SEM Tian = Tianeptine.

We previously reported that tianeptine enhances submaximal theta burst-induced LTP in mouse CA1 pyramidal neurons and rescues impaired LTP caused by prior acute stress, or corticosterone (12). We now examined if tianeptine enhancement of eEPSCs involves common target(s) and signaling pathways to those implicated in LTP. eEPSCs were evoked by stimulating two spatially separated synaptic inputs (Str. radiatum and Str. oriens) innervating the apical and basal dendrites of a CA1 pyramidal neuron respectively (Fig. 3*A*). Upon obtaining stable control eEPSCs, for both pathways, at 5 min one input (Str. radiatum) was given a pairing protocol (duration 3 min) as we described (37), resulting in potentiation (LTP) of the eEPSC amplitude (determined at 13 to 18 min =1.64 ± 0.11; n = 7), while the control input (Str. oriens) remained unchanged (1.07 ± 0.01; n = 7; Fig. 3 *B*–*D*). Following establishment of LTP, the subsequent application of tianeptine (50 µM) did not further enhance the potentiated pathway (33 to 38 min = 1.63 ± 0.06; n = 7), but in the same slice increased the eEPSC amplitude evoked via the control input (1.46 ± 0.04; n = 7; Fig. 3 *B*–*D*). The lack of effect following pairing may indicate engagement of common receptor targets and/or intracellular biochemical machinery by tianeptine and LTP. Phosphorylation of GluA1-AMPARs at GluA1(S831) by calcium/calmodulin kinase II (CaMKII) is an important step in LTP induction (38). Acute tianeptine increases phosphorylation of the GluA1(S831) residue and additionally the GluA1(S845) residue, a protein kinase A (PKA) substrate (11, 39). To explore CaMKII involvement, we compared the effects of KN-93 upon LTP and tianeptine enhancement of eEPSCs. KN-93 binds to calmodulin disrupting the interaction of calcium/calmodulin with CaMKII, thereby indirectly inhibiting CaMKII activation (40). With intracellular KN-93 (10 µM in the recording pipette) the pairing protocol did not trigger LTP (1.00 ± 0.07; n = 11; Fig. 3 *E*–*G*) and the effect of tianeptine (50 µM) on the eEPSC amplitude was completely blocked (1.0 ± 0.03; n = 8; Fig. 3 *H*–*J*). As a control, intracellular KN-92 (10 µM), an inactive KN-93 analog, had no influence on the effect of tianeptine on the eEPSC amplitude (1.8 ± 0.01, n = 6; Fig. 3 *H*–*J*), and in contrast to KN-93, KN-92 has no effect on CA1 LTP (41). These data suggest phosphorylation of GluA1-AMPARs by CaMKII is important for LTP induction and for tianeptine enhancement of AMPAR function. Collectively, these experiments reveal similarities between tianeptine and this form of LTP on eEPSCs. Hippocampal CA1 LTP requires NMDAR-mediated Ca^2+^ entry. However, the NMDAR antagonist AP-5 (100 µM), had no effect on the tianeptine (50 µM) enhancement of the eEPSC amplitude (1.95 ± 0.17 n = 6 neurons), distinguishing tianeptine from “physiological” Ca^2+^-induced LTP (Fig. 3 *H*–*J*).

### Tianeptine Facilitation of Synaptic AMPARs Is Mediated Indirectly by Opioid Receptor Activation and Is Mimicked by Opioid Agonists.

**Fig. 4.**
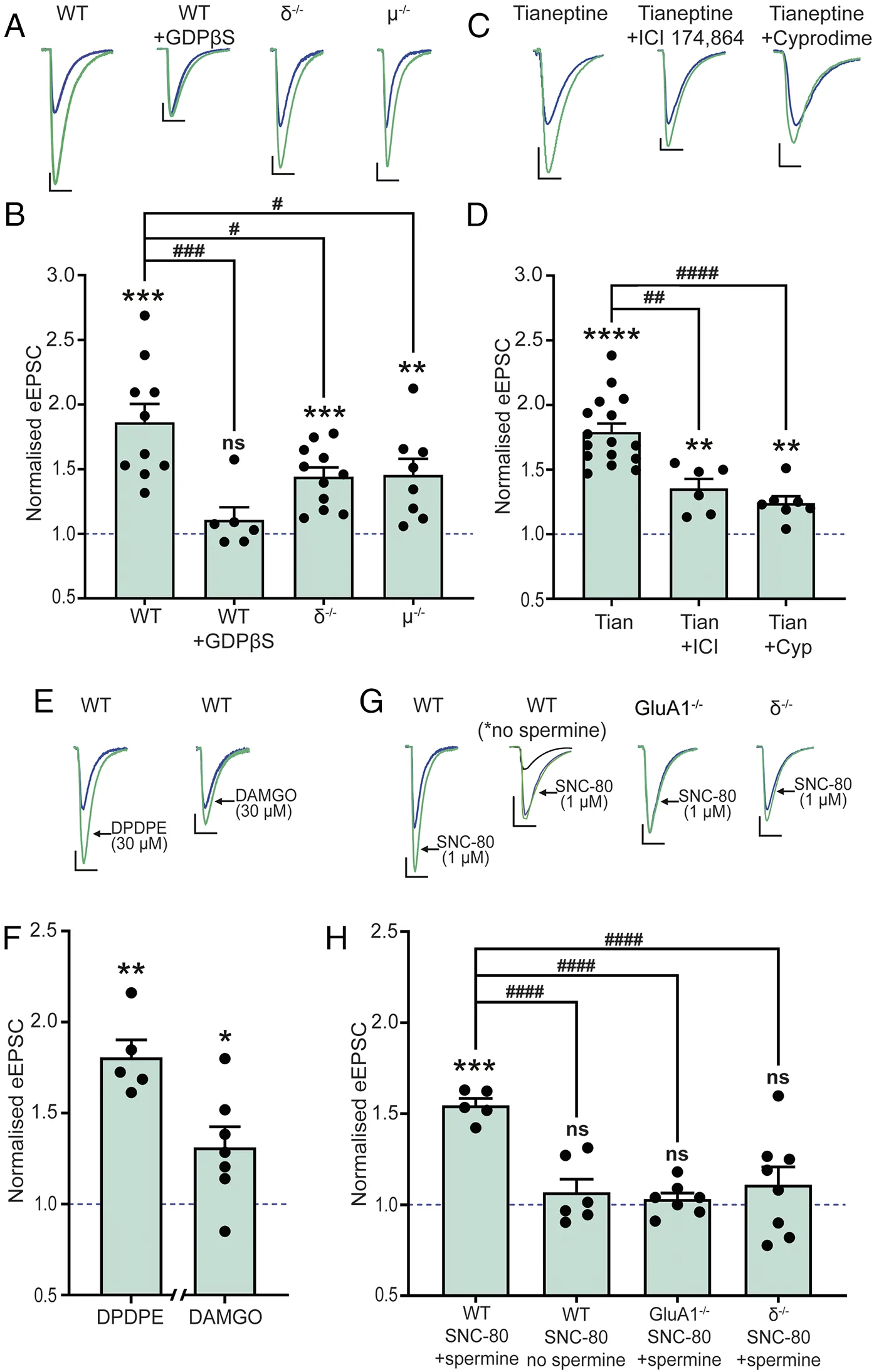
Opioid receptors mediate the enhancement of hippocampal glutamatergic neurotransmission by tianeptine and by opioids. (*A*) Representative traces illustrating an ensemble average of eEPSCs recorded from CA1 pyramidal neurons (with intracellular spermine, 100 µM) obtained from WT mice in the i) absence and ii) presence of intracellular GDPβS (0.5 mM), iii) from δ-receptor (δ^–/–^) and iv) µ-receptor (µ^–/–^) knockout mice, in each case before (blue trace) and 10 min after tianeptine (50 µM) perfusion (green trace). (*B*) A histogram comparing the impact of treatment (±GDPβS) and of genotype (δ^–/–^ and µ^–/–^) on the effect of tianeptine (50 µM) upon the eEPSC peak amplitude. A one-way ANOVA identified a significant difference between the groups (F(3, 31) = 6.7, *P* < 0.01). In comparison to WT neurons a Dunnett’s multiple comparison post hoc analysis revealed the effect of tianeptine to be significantly reduced for WT CA1 neurons in the presence of intracellular GDPβS (### = *P* < 0.001) and for δ^–/–^ (# = *P* < 0.05) and µ^–/–^ (# = *P* < 0.05) neurons. Subsequent one-sample *t* tests demonstrated that tianeptine produced a significant increase of the eEPSC amplitude for all genotypes (WT = 1.86 ± 0.14, n = 10, 7 mice, *** = *P* < 0.001; δ^–/–^ = 1.44 ± 0.07; n = 11, 5 mice, *** = *P* < 0.001; µ^–/–^ = 1.46 ± 0.12, n = 8, 4 mice, ** = *P* < 0.01), but had no significant effect (ns) upon WT neurons with intracellular GDPβS (1.11 ± 0.10, n = 6, 3 mice *P* > 0.05). (*C*) Representative traces of averaged eEPSCs recorded from CA1 pyramidal neurons (with intracellular spermine, 100 µM) obtained from WT mice before (blue traces) and after 50 µM tianeptine alone, tianeptine coapplied with 10 µM of the δ-opioid receptor antagonist ICI 174,864 or with 20 µM of the µ-opioid receptor antagonist cyprodime (green traces). (*D*) A histogram investigating the influence of ICI 174,864 (10 µM) and cyprodime (20 µM) on the effect of tianeptine on eEPSC amplitude. A one-way ANOVA revealed a significant effect of treatment (F(3,31) = 12.0; *P* < 0.0001). A Dunnett’s multiple comparison post hoc test demonstrated the effect of tianeptine (1.8 ± 0.07, n =16, 10 mice) to be significantly reduced by ICI 174,864 (## = *P* < 0.01, 1.4 ± 0.07, n = 6, 3 mice), and by cyprodime (#### = *P* < 0.0001; 1.2 ± 0.05, n = 7, 3 mice). The effect of tianeptine was significantly reduced by either antagonist, although further analysis (a one-sample *t* test) revealed that tianeptine significantly increased the eEPSC amplitude in the absence (tianeptine alone **** = *P* < 0.0001) and in the presence of the antagonists (tianeptine + ICI 174,864: ** = *P* < 0.01; tianeptine + cyprodime: ** = *P* < 0.01). (*E*) Representative traces illustrating averaged eEPSCs recorded (with intracellular spermine, 100 µM) from WT mouse neurons before (blue trace) and 10 min after (green trace) application of the selective δ- and µ-receptor agonists DPDPE (30 µM) and DAMGO (30 µM) respectively. (*F*) A histogram quantifying the effects of these peptides upon the eEPSC peak amplitude of WT mouse neurons. DPDPE (30 µM) significantly increased the eEPSC amplitude (1.81 ± 0.10 relative to control, ** = *P* < 0.01 one-sample *t* test; n = 5, 3 mice). Similarly, DAMGO (30 µM) had a significant, albeit modest effect upon the eEPSC amplitude (1.31 ± 0.11 relative to control, * = *P* < 0.05 one-sample *t* test, n = 7, 3 mice). (*G*) Illustrated are representative traces of averaged eEPSCs before (blue trace) and 10 min after (green trace) the application of the selective δ-receptor agonist SNC-80 (1 µM). Note the SNC-80 no spermine traces include an eEPSC trace (black) recorded shortly after breaking into the neuron. The recordings were obtained from WT neurons either in the presence or absence of intracellular spermine (100 µM) as indicated. The additional recordings illustrated were from neurons derived from *Gria1^–/–^* (GluA1^−/−^) and δ^–/–^ (δ^−/−^) mice (in the presence of intracellular spermine 100 µM). (*H*) A histogram illustrating the influence of the condition (±spermine 100 µM) and genotype (*Gria1^–/–^*, δ^–/–^) upon the effect of SNC-80 (1 µM) on the eEPSC amplitude. A one-way ANOVA revealed a significant effect of group (F(4, 31) = 13.5, *P* < 0.0001) and a Dunnett’s multiple comparison post hoc test revealed a significant difference between the WT with intracellular spermine and each of the other groups (#### = *P* < 0.0001). Subsequent analysis (one-sample *t* test) demonstrated SNC-80 (1 µM) to produce a significant enhancement of the eEPSC amplitude of WT neurons with intracellular spermine (1.55 ± 0.04; *** = *P* < 0.001; n = 5, 4 mice), but with no significant effect on the eEPSC amplitude of WT neurons in the nominal absence of this polyamine (1.07 ± 0.07; ns = *P* > 0.05; n = 6, 3 mice). Additionally, SNC-80 had no significant effect upon the eEPSC amplitude of neurons derived from the *GluA1^–/–^* mouse (1.03 ± 0.03; ns = *P* > 0.05; n = 7, 4 mice), or the δ^–/–^ mouse (1.11 ± 0.10 of control, ns = *P* > 0.05, n = 8, 6 mice). For all histograms (*B*, *D*, *F*, and *H*) each column represents the mean ± SEM. For panels *A*, *C*, *E*, and *G* the eEPSC calibration bars are y-axis = 50 pA, x-axis = 10 ms. Tian = tianeptine, ICI = ICI 174,864, Cyp = cyprodime.

KN-93 impairment of the tianeptine facilitation of the eEPSC suggests an indirect effect on GluA1-AMPARs. The eEPSC amplitude increase by tianeptine (50 µM) was prevented by intracellular inclusion of 0.5 mM GDP-βS in the recording pipette (1.11 ± 0.1; n = 6), demonstrating a requirement for postsynaptic G-proteins (Fig. 4 *A* and *B*). Tianeptine is an agonist of µ- and δ-receptors (3, 6). In agreement, tianeptine activated human recombinant µ- and δ-receptors expressed in HEK293 cells, thereby reducing forskolin-stimulated cAMP levels (IC_50_ = 1.7 ± 0.27 µM; n = 10; IC_50_ = 8.7 ± 1.58 µM; n = 20) respectively (*SI Appendix*, Fig. S2*A*). In keeping with a requirement for opioid receptors, untransfected HEK293 cells did not respond to tianeptine (1 nM to 100 µM; n = 5)—(*SI Appendix*, Fig. S2*A*). We next compared the influence of tianeptine upon WT neurons with those obtained from mice lacking either μ- or δ-receptors (µ^–/–^ and δ^–/–^ mice, respectively). Tianeptine (50 µM) increased the eEPSC amplitude for all genotypes, but the effect was reduced for neurons obtained from either the δ^–/–^ or the µ^–/–^ mice (WT = 1.86 ± 0.14; n = 10; δ^–/–^ = 1.44 ± 0.07; n = 11; µ^–/–^ = 1.46 ± 0.12; n = 8; Fig. 4 *A* and *B*). Complementing these findings the effect of tianeptine on WT neurons (1.8 ± 0.07, n = 16) was significantly reduced by 10 µM coapplication of the δ-receptor antagonist ICI174,864 (1.4 ± 0.07, n = 6) and by 20 µM of the µ-receptor antagonist cyprodime (1.2 ± 0.05, n = 7; Fig. 4 *C* and *D*). Further linking opioid receptor activation and synaptic AMPARs the selective δ- and µ- receptor peptide agonists [D-Pen^2^,D-Pen^5^]enkephalin (DPDPE) (30 µM) and DAMGO (30 µM) respectively increased the eEPSC amplitude (DPDPE = 1.81 ± 0.10; n = 5) and (DAMGO =1.31 ± 0.11; n = 7; Fig. 4 *E* and *F*). Peptide penetration into the slice may be compromised by endogenous peptidases, leading to an underestimation of their efficacy (43). SNC-80, a nonpeptide δ-receptor-selective agonist, reduced cAMP accumulation in δ-receptor expressing HEK293 cells (IC_50_ = 1.7 ± 0.35 nM, n = 6), whereas much greater concentrations (≥300 µM) had no effect upon cells expressing the µ-receptor (n =10; *SI Appendix*, Fig. S2*B*). Given this selectivity, we investigated the effect of SNC-80 upon eEPSCs. SNC-80 (1 µM), in the presence of intracellular spermine, produced an increase of the eEPSC peak amplitude (1.55 ± 0.04; n = 5), but was inert in the absence of added spermine (1.07 ± 0.07; n = 6; Fig. 4 *G* and *H*). Furthermore, with intracellular spermine, SNC-80 (1 µM) had no effect upon the eEPSC amplitude recorded from CA1 neurons of *Gria1^–/–^* mice (1.03 ± 0.03; n = 7; Fig. 4 *G* and *H*). Therefore, in common with tianeptine, the synaptic AMPAR enhancing effects of SNC-80 are spermine-dependent and selective for GluA1-AMPARs. Complementing the δ-receptor selectivity in the cAMP assay, SNC-80 (1 µM) had no effect upon the eEPSC amplitude of CA1 neurons obtained from δ^–/–^ mice (1.11 ± 0.1; n = 8; Fig. 4 *G* and *H*).

## Discussion

The discovery that tianeptine activates µ- and δ-receptors identified credible targets whereby this elusive, atypical antidepressant may influence mood and memory (3, 6, 44). Our study revealed tianeptine and opioids activate hippocampal postsynaptic µ-and δ-receptors, thereby facilitating glutamatergic transmission mediated by synaptic GluA1-AMPARs. These effects may contribute to the behavioral effects of tianeptine and more broadly to those of endogenous and synthetic opioids.

### Activation of Hippocampal Opioid Receptors Facilitates Synaptic GluA1-AMPAR Function.

Acute tianeptine enhanced glutamatergic transmission by relieving the block of GluA1-AMPARs by endogenous polyamines (*SI Appendix*, Fig. S3). In support, in the nominal absence of intracellular polyamines in the pyramidal neuron, tianeptine had no effect on the eEPSC amplitude, implying a postsynaptic locus. Furthermore, the effect of tianeptine was reversed by extracellular NASPM. For AMPARs polyamine block is selective for CP-AMPARs and in agreement tianeptine was inactive on CA1 neurons obtained from the *Gria1^–/–^* mouse.

In contrast to hippocampal slices, tianeptine had no effect on the phosphorylation of GluA1 subunits (GluA1-Ser831; GluA1-Ser845) when expressed in control HEK293 cells, implying these non-neuronal cells lack components required for tianeptine action (11). Previous biochemical studies, replicated here, reported tianeptine to activate G-protein-coupled µ- and δ-receptors when expressed in HEK-293 cells (3). Functionally, the effect of tianeptine to enhance the eEPSC amplitude was completely prevented by postsynaptic inclusion of the G-protein inhibitor GDP-βS. In recordings from CA1 pyramidal neurons, deletion of either the µ- or the δ-receptor, or their pharmacological antagonism by cyprodime or ICI174,864 reduced, but did not abolish, the tianeptine-induced enhancement of the eEPSC, consistent with a partial overlap and convergence of μ- and δ-receptor signaling onto a common postsynaptic GluA1-AMPAR mechanism. The antagonist effects provide pharmacological evidence for receptor involvement, rather than a quantitative comparison of receptor subtype contribution, as their impact is influenced by concentration, receptor occupancy, and slice penetration. Nevertheless, the ability of μ- and δ-selective agonists (DAMGO and DPDPE respectively) to enhance eEPSCs, together with the δ-receptor-dependent action of SNC-80, indicates that at least in these neurons, activation of either receptor class is sufficient to engage this AMPAR-modulatory pathway. Thus, for CA1 pyramidal neurons our findings support a model in which μ- and δ-receptors make partially overlapping contributions to the tianeptine-induced facilitation of GluA1-AMPAR-mediated transmission.

How opioid receptors influence GluA1-AMPAR function requires further investigation. Opioid receptors engage various downstream signaling pathways. In cell lines, selective activation of µ- or δ-receptors rapidly phosphorylates MAP kinase (45). Functionally, facilitation of hippocampal fEPSPs by tianeptine is blocked by MAP kinase inhibition and by inhibitors of PKA and CaMKII (11, 12). As an initial step toward unraveling the signaling pathway we focused on Calmodulin/CaMKII as 1) it influences the end target, the GluA1-AMPAR by phosphorylating the GluA1 serine 831 residue; 2) in mouse brain slices tianeptine increases phosphorylation of this residue and additionally S845, a PKA substrate, whereas in rats it selectively phosphorylates S831 (11, 22). Phosphorylation of S831 enhances GluA1-AMPAR responses by increasing the proportion of large conductance single channel openings, whereas phosphorylation of S845 increases open probability, effects that may contribute to the increased eEPSC amplitude by tianeptine. Tianeptine phosphorylates CaMKII (11) and here the facilitation of eEPSCs by this antidepressant was abolished by indirectly inhibiting CaMKII activation with intracellular KN-93 (40), but not by the inactive analog KN-92. Complementing this rapid facilitation of synaptic AMPAR function, tianeptine also influences hippocampal neuron AMPAR location by reducing their surface diffusion, predominantly stabilizing synaptic occupancy (12). This trafficking effect also involves CaMKII, which facilitates the binding of PSD-95 with the phosphorylated AMPAR auxiliary subunit stargazin [TARP, γ2] (12). The involvement of transmembrane AMPAR regulatory proteins (TARPs, mainly γ8 in the hippocampus) may be pertinent to the immediate effect of tianeptine on eEPSCs as TARPs influence polyamine block of CP-AMPARs (32, 46). Whether these AMPAR trafficking effects contribute to the rapid impact of tianeptine on eEPSCs, an effect that commences immediately the drug perfuses the tissue, is not known.

Activation of hippocampal astrocytic µ-receptors by DAMGO is reported to increase glutamate release from astrocytes, which in turn activates presynaptic mGluR1s to increase vesicular glutamate release (47). However, tianeptine has no effect on paired pulse facilitation of fEPSPs (11) and here had no effect on the sEPSC frequency. Additionally, the essential requirement for intracellular spermine and the inhibitory effect of intracellular GDP-βS further implicates a postsynaptic locus for the effects of tianeptine and SNC-80 on synaptic AMPARs and suggests the involvement of opioid receptors located on CA1 pyramidal neurons (*SI Appendix*, Fig. S3). Although both µ- and δ-receptors are primarily expressed on hippocampal GABA-ergic interneurons, the presence of δ-receptor mRNA and protein has been reported in rodent hippocampal glutamatergic principal neurons (48–51).

Both ampakines and tianeptine increase brain derived neurotrophic factor (BDNF) levels and tianeptine influences neuronal BDNF trafficking (18, 52). The effect of tianeptine on BDNF transport is blocked by a TRK-B/BDNF receptor antagonist, suggesting tianeptine, in common with the active ketamine metabolite (2R, 6R)-HNK and LSD, agents that also exhibit rapid antidepressant-like activity, interacts with the TRK-B receptor (52–54). Like tianeptine, BDNF activation of TRK-B receptors increases phosphorylation of GluA1 S831 and influences hippocampal AMPAR mobility (52, 55). Whether directly, or via BDNF, such AMPAR plasticity may contribute to the maintained effects of tianeptine upon mood and cognition. Note δ-receptor agonists including leu-enkephalin and SNC-80 also increase BDNF mRNA levels (28, 56, 57).

### Tianeptine, Opioid Receptors, AMPARs, and Depression.

Does facilitation of glutamatergic transmission via activation of opioid receptors contribute to the antidepressant and memory enhancing effects of tianeptine? The hippocampus is a pertinent locus, being implicated in mood disorders, learning and memory and local administration of tianeptine into the mouse ventral hippocampus produces antidepressant-like effects (6, 58). Clinical and preclinical evidence implicate perturbations of glutamatergic transmission in depression (14). Developing antidepressants that enhance AMPAR function has received considerable attention (15, 18, 19). AMPAR positive allosteric modulators (PAMs) exhibit antidepressant-like behavior in rodent models of mood disorders (18). The AMPAR PAM, NBI-1065845, (formerly TAK-653) is undergoing clinical trials to treat major depressive disorder (17). Most AMPAR PAMs slow receptor desensitization or reduce receptor deactivation and exhibit limited selectivity across AMPAR subtypes (18). By contrast, at least in CA1 pyramidal neurons, the AMPAR effect of tianeptine is selective for the GluA1-AMPAR isoform and requires expression of µ- or δ-receptors. Consequently, the profile of tianeptine may be distinct from other AMPAR PAMs.

Implicating GluA1-AMPARs in mood disorders and as a target for antidepressants, *Gria1^–/–^* mice exhibit increased helplessness behavior in an aversive foot shock model of depression (59). The GluA1(S831A) phospho-resistant mutant mouse exhibits a reduced sucrose preference and impaired performance in a novelty suppressed feeding task, suggesting a depressive-like phenotype (13). Tianeptine increased phosphorylation of the GluA1Ser831/Ser845 residues (5, 11) and importantly the effects of this antidepressant to reduce immobility in the tail suspension test and to enhance locomotion were impaired in mice with the phospho-resistant GluA1(S831A) mutation (5). Associating GluA1-AMPARs with conventional antidepressants, the 5-HT uptake blocker fluoxetine restored sucrose preference in WT mice subjected to prior chronic unpredictable stress but had no effect on the reduced sucrose preference of the GluA1(S831A) mouse (13). Complementing these findings in vitro studies revealed fluoxetine to produce an acute enhancement of fEPSPs evoked by stimulation of the temporoammonic pathway, an effect indirectly mediated by activation of postsynaptic 5-HT1B receptors, blocked by inhibition of CaMKII and not evident in the GluA1(S831A) mouse (13).

Opioid receptors also present a viable target to mediate the antidepressant effects of tianeptine. Endogenous and synthetic opioid agonists have long been implicated in mood disorders. Enkephalins, endorphins, and enkephalinase inhibitors exhibit antidepressant-like effects in rodent despair paradigms (26–29). In mice genetic deletion of the δ-, but not the µ-receptor promotes anxious and depressive-like behaviors, suggesting an influence of endogenous opioid peptides upon mood (27, 28). In a chronic stress mouse model, chronic fluoxetine increased proenkephalin levels and improved performance in the forced swim and novelty suppressed feeding tests, in both WT and µ-KO mice but not in δ-KO mice, a result that may reflect the selectivity of enkephalins for δ-receptors (60). Selective δ-receptor agonists have attracted attention as putative antidepressants. In rodents they decrease despair associated behaviors in various paradigms and have undergone clinical trials (26–28, 61). By contrast, rodent studies to date primarily implicate µ-receptors in the antidepressant-like effects of tianeptine. Tianeptine was ineffective in reducing immobility in the mouse forced swim test in the µ-receptor knockout mouse, and when the µ-receptor was selectively antagonised by cyprodime, but remained efficacious in the δ-receptor knockout mouse (3, 6, 44). In WT, but not in µ-receptor knockout mice tianeptine was active in two additional depression associated paradigms (novelty suppressed feeding and chronic odor restraint stress) (6).

In CA1 pyramidal neurons activation of µ- and δ-receptors by tianeptine produced similar effects on the eEPSC amplitude. Despite this equivalence, in antidepressant studies of tianeptine the δ-receptors in the µ-receptor knock out (KO) mouse do not appear to compensate, potentially challenging the behavioral relevance of the AMPAR-enhancing effects mediated by δ-receptors. However, in vivo the lower affinity of tianeptine for the δ- vs. the µ-receptor may be important. Furthermore, CA1 principal neurons in the dorsal hippocampus may not be the relevant locus and are unlikely to be the sole location for the antidepressant-like actions of tianeptine (6) but may be important for improving memory (62). Indeed, we find that tianeptine greatly improves performance of WT mice in a hippocampal-dependent spatial-memory-based cross-maze task but is inert in both the µ- and the δ-receptor KO mice (63).

Adding complexity regarding the mechanism of tianeptine action, opioid receptors are also expressed presynaptically e.g., on hippocampal interneurons, which upon activation decrease vesicular gamma-aminobutyric acid (GABA) release (*SI Appendix*, Fig. S3). Such disinhibition is emerging as a credible mechanism of certain rapidly acting antidepressants e.g., ketamine (64, 65). Indeed, the selective deletion of µ-receptors from somatostatin- but not parvalbumin-GABAergic interneurons impaired the acute effect of tianeptine in the forced swim test (6). Activation by [D-Ala^2^, N-MePhe^4^, Gly-ol^5^]-enkephalin (DAMGO) of presynaptic µ-receptors located upon CA1 hippocampal GABA-ergic interneurons decreased neurally evoked GABA release onto pyramidal neurons. (66). Similarly, we found tianeptine (50 µM) reduced the amplitude of neurally evoked inhibitory postsynaptic currents (eIPSCs) and the frequency of spontaneous IPSCs (sIPSCs) (67). The dual engagement of both pre- and postsynaptic opioid receptors provides a powerful mechanism to influence the neuronal excitatory/inhibitory (E-I) balance and may contribute to the behavioral actions of tianeptine (*SI Appendix*, Fig. S3).

### The Procognitive Effects of Tianeptine.

Unusually for an antidepressant, tianeptine exhibits procognitive properties. Memory dysfunction is prevalent in over 50% of patients suffering from MDD, having a particular impact on encoding and episodic memory retrieval, implicating the hippocampus (8, 9). Additionally, a recent study on Alzheimer’s disease patients reported tianeptine to improve mood and enhance memory performance (68). Stressful events may predispose to depression and many preclinical models of mood disorders are based on the impact of prior acute or chronic stress on control animals (4, 12, 15, 69, 70). In rodents, tianeptine rescued impaired hippocampal and prefrontal cortex LTP caused by prior acute stress (4, 12, 69, 70) and prevented the effect of the stress hormone corticosterone to increase AMPAR diffusion in hippocampal neurons (12). Furthermore, in mouse models of Huntington disease chronic treatment with tianeptine both rescued the impaired hippocampal LTP and improved performance in hippocampal-dependent behavioral tasks (52). As described above, implicating opioid receptors, the effects of tianeptine in a hippocampal spatial memory task were prevented in both µ- and δ-receptor KO mice (63).

Distinct from LTP, the effects of tianeptine upon eEPSCs did not require NMDAR participation, although some mechanisms appear common. Prior LTP prevented the effects of subsequent tianeptine, whereas in the same hippocampal slice tianeptine enhanced eEPSCs resulting from stimulating a naïve control pathway. The AMPAR enhancing effect of tianeptine requires the GluA1 subunit and in mice lacking this subunit, hippocampal CA1 LTP is impaired (25). GluA1-AMPAR phosphorylation by CaMKII (GluA1S831) and PKA (GluA1S845) is implicated in CA1 LTP (38, 39) and acute tianeptine increased phosphorylation of these residues (11). Here, both LTP and the acute effects of tianeptine upon eEPSCs were impaired by indirectly inhibiting CAMKII with KN-93. Furthermore, PKA inhibition was reported to block the effects of tianeptine upon fEPSPs (11). It is conceivable that prior phosphorylation of these GluA1 residues following LTP occludes the effect of subsequent tianeptine upon eEPSCs. Both LTP and tianeptine produce changes to synaptic AMPAR expression and location (12, 52, 71). Additionally, a suppression of GABA release from GABA-ergic interneurons may contribute to the effects of tianeptine upon LTP and cognition (6, 67). Finally, the effect of tianeptine upon BDNF expression and trafficking may be relevant given the BDNF effects to facilitate CA1 LTP (72).

In summary, activation of hippocampal postsynaptic opioid receptors by tianeptine selectively enhanced the function of GluA1-AMPARs by relieving their resident polyamine block (*SI Appendix*, Fig. S3). This rapid effect of tianeptine upon excitatory transmission may elicit slower and more maintained changes in other factors such as levels of BDNF and neurogenesis on a time scale that better mirrors the onset of antidepressant efficacy. Furthermore, this dialogue between opioid receptors and synaptic AMPARs may have broader implications, by contributing to the effects of endogenous and administered opioids upon neuronal signaling and behavior.

## Materials and Methods

Experiments were performed in Dundee, Heidelberg, and Moscow. Investigation of the effect of tianeptine on sEPSCs and exploring the role of opioid receptor subtypes in mediating the effects of tianeptine and opioid receptor agonists upon eEPSCs were conducted in Dundee. Studies with WT (C57BL/6J), µ- and δ-receptor knockout mice (p18-26), were performed in accordance with UK Home Office guidelines and regulations using approved procedures (Home Office License number PP4945903). All experimental protocols were approved by both the University of Dundee’s ethical review committee and the Home Office. Mouse lines were fully backcrossed onto the C57BL/6J background obtained from Jackson Labs. The µ- and δ-receptor knockout mice were obtained from Jackson Labs (strain #007559; B6.129S2-*Oprm1tm1Kff/J* and strain #007557; B6.129S2-*Oprd1tm1Kff/J* respectively). The C57BL/6J WT mice (Charles River, Kent, UK) were periodically bred with knockout mice and genotyped using genomic DNA sent to Transnetyx (Cordova, TN), as we previously described (73). Mice were maintained, bred, and killed humanely in accordance with UK Home Office regulations.

In Heidelberg the GluA1 knock-out mice (*Gria1^–/–^* mice) were generated as described (25, 30) under the license of the Governmental Council Karlsruhe, Germany (35-9185.81/G-4/02). Animal care was compliant with the institutional guidelines at the animal facilities of the Center of Molecular Biology (ZMBH, INF 282, D-69120 Heidelberg, Germany) and the Max Planck Institute for Medical Research in Heidelberg under the approval of the Animal Care Committee at the Governmental Council Karlsruhe, Germany: Generation of mice (35-9185.81/G-4/02). Heterozygous *Gria1* mice (25, 30) were routinely intercrossed and crossed to C57BL/6J mice (Charles River) to generate cohorts of experimentally naïve mice and WT controls (25, 30). In Moscow the study with WT mice was approved by the Independent Ethical Committee of the Federal Center of Brain Research and Neurotechnologies, Moscow, Russia (#116/10.06.2025).

### Brain Slice Preparation.

In Dundee, experimental protocols using whole-cell voltage-clamp of mouse CA1 pyramidal neurons were adapted from our previous publication (74). Mice (P16-24) of either sex were killed by cervical dislocation. The brain was dissected and placed into semifrozen oxygenated (95% O_2_, 5% CO_2_) artificial cerebrospinal fluid (aCSF) containing the following (in mM): 234 sucrose, 2.5 KCl, 1.25 NaH_2_PO4, 10 MgSO_4_, 0.5 CaCl_2_, 26 NaHCO_3_, 10 glucose (340 to 342 mOsm). Using a Leica VT1200S vibratome (Leica, Nussloch, Germany), sagittal slices were cut at a thickness of 350 μm at 0 to 4 °C, then subsequently transferred to a holding chamber with oxygenated (95% O_2_, 5% CO_2_) extracellular solution (ECS) containing (in mM): 126 NaCl, 26 NaHCO_3_, 10 glucose, 2.5 MgCl_2_, 2.95 KCl, 1.25 NaH_2_PO_4_, 2.5 CaCl_2_ (305 to 310 mOsm, pH 7.4) and maintained at room temperature for 1 h prior to being transferred to the recording chamber.

### Whole-Cell Voltage-Clamp Recordings.

The hippocampal CA1 region was identified using an Olympus microscope equipped with DIC/IR optics. Individual pyramidal neurons embedded in the CA1 cell layer were identified using a 40× lens and a magnification chamber that allows an additional 1.6× magnification. Recording pipettes were pulled from fire-polished thick-wall borosilicate glass (1.55 mm ± 0.05 O.D., 0.95 mm ± 0.05 I.D., 75 mm ± 0.05, King Precision Glass, Inc., CA) employing a two-stage protocol with a Narashige PC-10 vertical electrode puller (Narashige, Japan). Recording pipettes (3 to 5 MΩ) were filled with ICS of the following composition (in mM): Cs-gluconate (105), CsCl (30), HEPES (10), EGTA (0.2), NaCl (8), Na_3_GTP (0.3), 4 Mg-ATP (4), disodium-phosphocreatine (7), (pH = 7.4). In the majority of experiments, the ICS was supplemented with 100 µM spermine. A “Patch Star” micromanipulator (Scientifica, UK) was used to lower the patch electrode into the recording chamber. The hippocampal slice was maintained by perfusion of the ECS (described above), sustained at 29 to 30°C, bubbled with a 95% oxygen/ 5% CO_2_ mix, supplemented with gabazine (10 µM) to inhibit GABA_A_ receptors and in all the experiments in Dundee, AP5 (50 µM) was included to inhibit NMDA receptors.

Whole-cell voltage-clamp recordings were obtained using an Axopatch 1D amplifier (Molecular Devices, CA). Recordings were made at a holding potential (Vh) of −70 mV. Upon obtaining the whole-cell configuration, the neuronal capacitance and series resistance were adjusted. If the series resistance changed by more than 20% during acquisition the recordings were discarded. Membrane currents were filtered at 2 kHz (8-pole, low-pass Bessel filter) and recorded directly to a computer using an A/D converter (NIDAQmx; National Instruments) at a 10 kHz sampling rate. Additionally, recordings were continuously monitored with a digital oscilloscope (Hitachi VC-6525). Neurally evoked eEPSCs were elicited by stimulating the Schaffer collateral pathway at a frequency of 1/10 s. The duration of this biphasic stimulation was 40 µs and the magnitude of the stimulus delivered was adjusted to produce an eEPSC with an average amplitude of ~20% of the maximal eEPSC amplitude for that neuron.

The digitalized recordings of s- and e-EPSCs were analyzed offline using WinEDR and WinWCP software (Strathclyde University, UK). An algorithmic detection protocol was used for event detection in WinEDR. All events detected were then visually inspected within a 200 ms window to include any missed events, or to discard any electrical noise, incorrectly included in the algorithmic detection process using WinWCP. For all eEPSCs, an additional step of digitally removing the stimulation artefact before detection was performed in the WinWCP program. For sEPSCs at least 50 events from each neuron, for each recording condition, were collected and for eEPSCs at least 10 events were collected for each of the times specified. The peak amplitude was determined for individual sEPSCs and eEPSCs. The frequency of sEPSCs was determined over a representative 120 s window in the absence of electrical stimulation for each cell by running an event detection protocol based upon the event rise time. In Heidelberg the LTP studies using a pairing protocol were performed using young adult WT mice (p35+) of either sex (42). The role of GluA1-AMPARs in mediating the effects of tianeptine and SNC-80 were investigated in WT and *Gria1^–/–^* mice aged p18-24. See previous publications for details of experiments performed in Heidelberg and Moscow (25, 30, 37, 75).

### Statistical Analysis—Electrophysiology.

All quantified results are expressed as an arithmetic mean ± SEM. A paired Student’s *t* test was performed to determine if tianeptine exerted a significant effect upon the sEPSC amplitude (pA) and frequency (Hz). Throughout n = the number of neurons recorded with the number of mice provided. For eEPSCs a one-sample *t* test assessed any significant effect of treatment and compared the change relative to control (normalized to 1). To evaluate statistical significance between two groups an unpaired Student’s *t* test was used, whereas a One-way ANOVA with the Dunnett’s multiple comparison test or a Bonferroni comparison was used appropriately when comparing multiple groups. All statistical analysis was performed using GraphPad Prism 10.

*Details of the pharmacological studies of recombinant µ- and δ-receptors are provided in supplementary Methods.*

### Drug Sources and Preparation.

Tianeptine Kemprotec Limited (Carnforth, UK). AP5, gabazine (helloBio), DPDPE, DAMGO, SNC-80, KN-92, NASPM, cyprodime, ICI 174,864 (Tocris), KN-93, GDPβS (Sigma). Drugs were made up as concentrated stock solutions in either distilled water (gabazine, AP5, tianeptine, cyprodime, NASPM), or in dimethyl sulfoxide (DAMGO, DPDPE, SNC-80, KN-93, KN-92, ICI 174,864) and subsequently diluted to the final concentration in ECS.

## Supplementary Material

Appendix 01 (PDF)

Dataset S01 (XLSX)

## Data Availability

Study data are included in the article and/or supporting information.
