# Supplementary material for: Enhancement of hippocampal synaptic AMPA receptor function by the antidepressant tianeptine and by opioid peptides
Source: Proc Natl Acad Sci U S A. 2026 Oct 7;123(41):e2521987123. doi: 10.1073/pnas.2521987123 (PMC13663814; doi:10.1073/pnas.2521987123)
Supplement: Supplementary file 1 [file pnas.2521987123.sapp.pdf]

PNAS

Supporting Information for

Enhancement of hippocampal synaptic AMPA receptor function by the antidepressant tianeptine and by opioid peptides.

Anna Mariano, Andrei Rozov, Grant D. Phillips, Sam Singleton, Daniel Baptista- Hon, Fliza Valiullina-Rakhmatulina, Rolf Sprengel, Rosamund Langston, Stephen J. Martin, Tim G. Hales, Jeremy J. Lambert.

Corresponding Author: Jeremy J. Lambert
Email: j.j.lambert@dundee.ac.uk

This PDF file includes:

Supporting Information text – Methods for Figure S2
Figure S1
Figure S2
Figure S3

Other supporting materials for this manuscript include the following:

Supporting Dataset
Note: Data for Figure S1 and Figure S2 are located in "Supporting Dataset" file.

Supporting Information Text

Methods for Figure S2

Pharmacological studies of recombinant μ- and δ-receptors.

The effect of tianeptine and SNC-80 to act as agonists of human recombinant μ and δ receptors was assessed in transiently transfected HEK293 cells. The cells (1×10^6 per 35-mm dish) were co-transfected with cDNAs encoding an intracellular cAMP sensor (pGloSensor-22F), together with cDNAs encoding either μ- or δ-receptors (2 μg, using lipofectamine-2000F in a ratio of 1:2.5). A parallel transfection with cDNA encoding eGFP (2 μg) was performed under identical conditions. GFP was visualized using fluorescence microscopy to confirm successful transfection. Control HEK293 cells were treated identically with the exception that they were not transfected with μ- or δ- receptor cDNA. Luminescence corresponding to intracellular cAMP concentration was measured 2 h. following addition of forskolin (30 μM) and luciferin (3 mM) in the dark at room temperature. Opioid receptor activation was assessed by exposing cells to increasing concentrations of tianeptine (1 nM – 30 μM) or SNC-80 (0.3 pM - 30 μM) in forskolin and luciferin containing buffer for 30 min. Luminescence was assessed on a GloMax Navigator luminometer using a 1 s integration time. The luminescence (proportional to cAMP levels) in presence of tianeptine or SNC-80 was established for control cells and cells expressing μ- or δ-receptors and plotted as the log agonist concentration (M) *vs* cAMP levels expressed as a percentage of control. A logistic function was fitted to the data to derive the IC_{50}, (converted to the positive logarithm of IC_{50} [pIC_{50}] to enable statistical comparisons) and minimum and maximum responses. The latter were used to establish estimates of agonist efficacy. Note n = the number of individual experiments/plates. A two-tailed unpaired t-test was used to compare the pIC_{50} and efficacy of tianeptine acting upon μ and δ receptors.

Figures

Fig. S1.

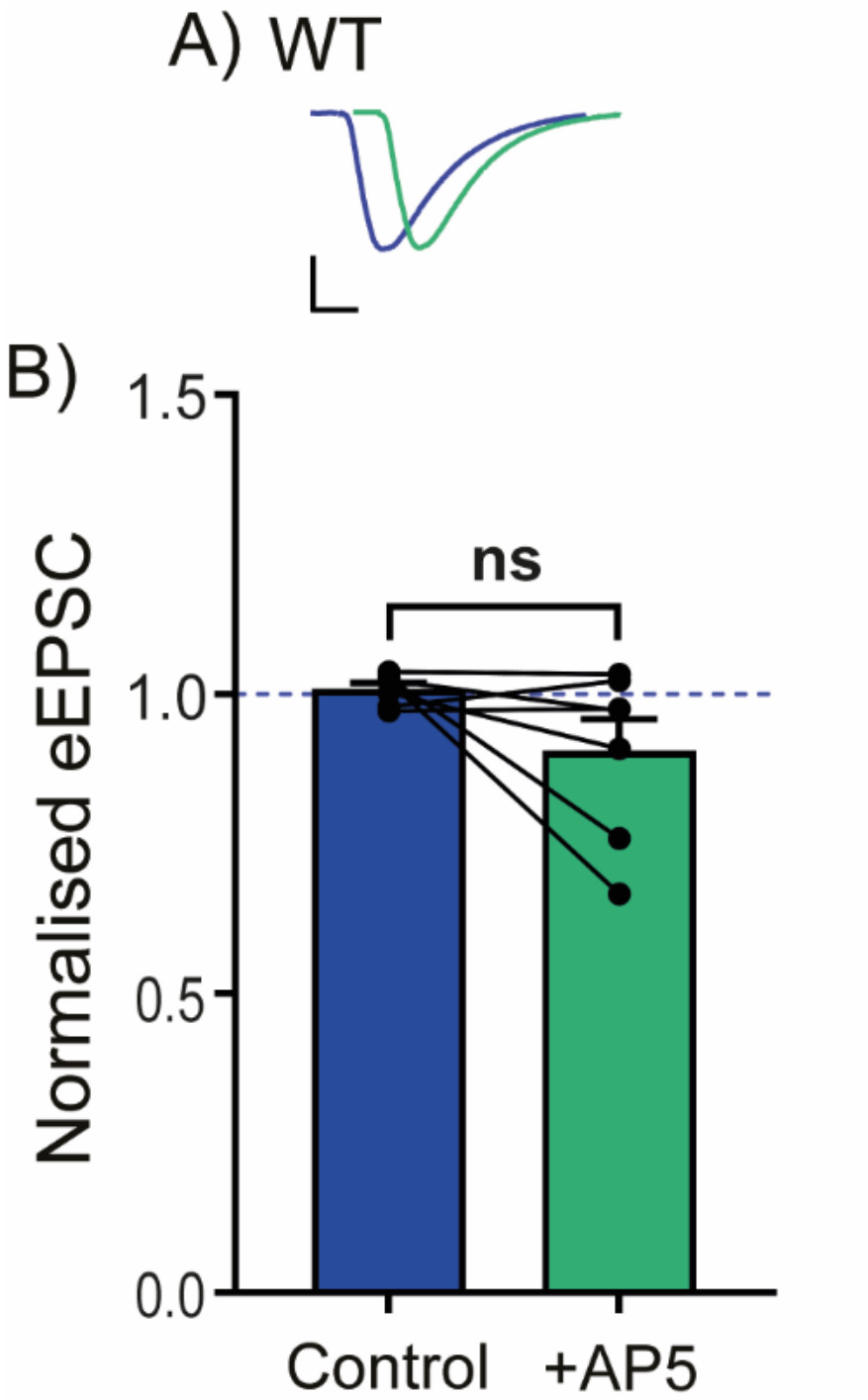

The NMDA receptor antagonist AP5 does not influence the amplitude of the neurally evoked EPSC. The eEPSCs were evoked by stimulation of the Schaffer collateral pathway once every 6s and the amplitude of 5 events were averaged into 30 s blocks over 5 min sections. ***A)*** *Traces illustrating an ensemble average of representative eEPSCs recorded from a CA1 pyramidal neuron (Vh = -70 mV extracellular 2.5 mM $MgCl_2$ 100 µM intracellular spermine) before (blue trace) and after 50 µM AP5 (green trace). Calibration bar 50 pA, 10 ms. B) The bar chart illustrates the control eEPSC amplitude (normalised to 1) determined 0-5 min. (blue) and following the bath application for 10-15 min of 50 µM AP5 (green). Under these recording conditions AP5 had no significant effect (ns) on the eEPSC amplitude. ($p > 0.05$; One Sample t-test).*

Fig. S2.

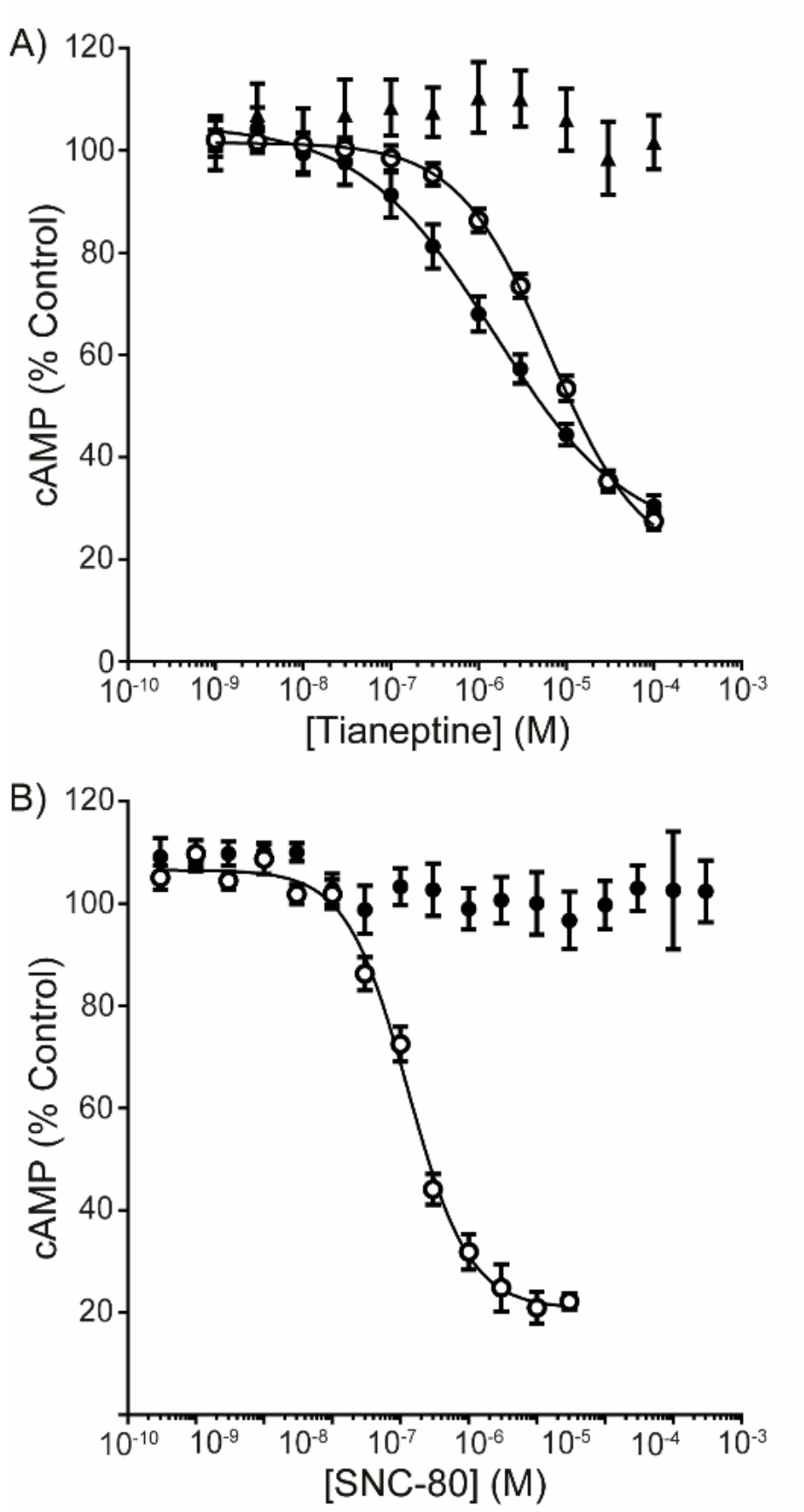

The interaction of tianeptine and of SNC-80 with μ- and δ-receptors. Concentration response relationships for tianeptine and SNC-80 on intracellular cAMP levels were assessed in HEK293 cells transiently expressing human μ- or δ-receptors and an intracellular cAMP sensor (pGloSensor-22F); (see Methods). The effect of **A)** tianeptine and **B)** SNC-80 upon luminescence corresponding to intracellular cAMP concentration was measured and plotted as the log of the agonist concentration *vs* cAMP levels expressed as a percentage of control. For tianeptine and SNC-80 each point represents the mean ± s.e.m for agonist activation of μ- (filled circles) or δ-receptors (open circles). A logistic function was fitted to the data to derive the Log IC_{50} converted to the pIC_{50} and to determine the efficacy of the agonist. **A)** In control HEK cells (*i.e.* not transfected with cDNAs for μ- or δ-receptors) tianeptine (10^{-9} – 10^{-4} M; filled triangles) had no effect on cAMP levels (filled triangles). By contrast, tianeptine produced a concentration dependent decrease of cAMP levels for HEK293 cells expressing the μ-receptor (filled circles) pIC_{50} = 5.8 ± 0.08 (n = 10) and for the δ-receptor (open circles) pIC_{50} = 5.1 ± 0.10 (n = 20). The difference in pIC_{50} values is significant, indicating that tianeptine is more potent as an agonist of μ-receptors compared to δ-receptors (p = 0.0001, two-tailed unpaired student's t-test). There was no significant difference in the efficacy of tianeptine acting upon μ-receptors (84 ± 3%) compared to δ-receptors (87 ± 5%) (p = 0.64, two-tailed unpaired student's t-test with Welch's correction). **B)** SNC-80 did not affect cAMP levels in cells transiently expressing μ-receptors (filled circles, n = 10), assessed over a wide range of concentrations (10^{-12} M – 10^{-4} M). A logistic function was fitted to the data representing SNC-80 interacting with δ-receptors (open circles) to derive a mean pIC_{50} value of 8.8 ± 0.07 and an efficacy of 88 ± 3% (n = 6). Note for both tianeptine and for SNC-80 each experiment was run in duplicate and each n number is a separate 96 well plate.

Fig. S3.

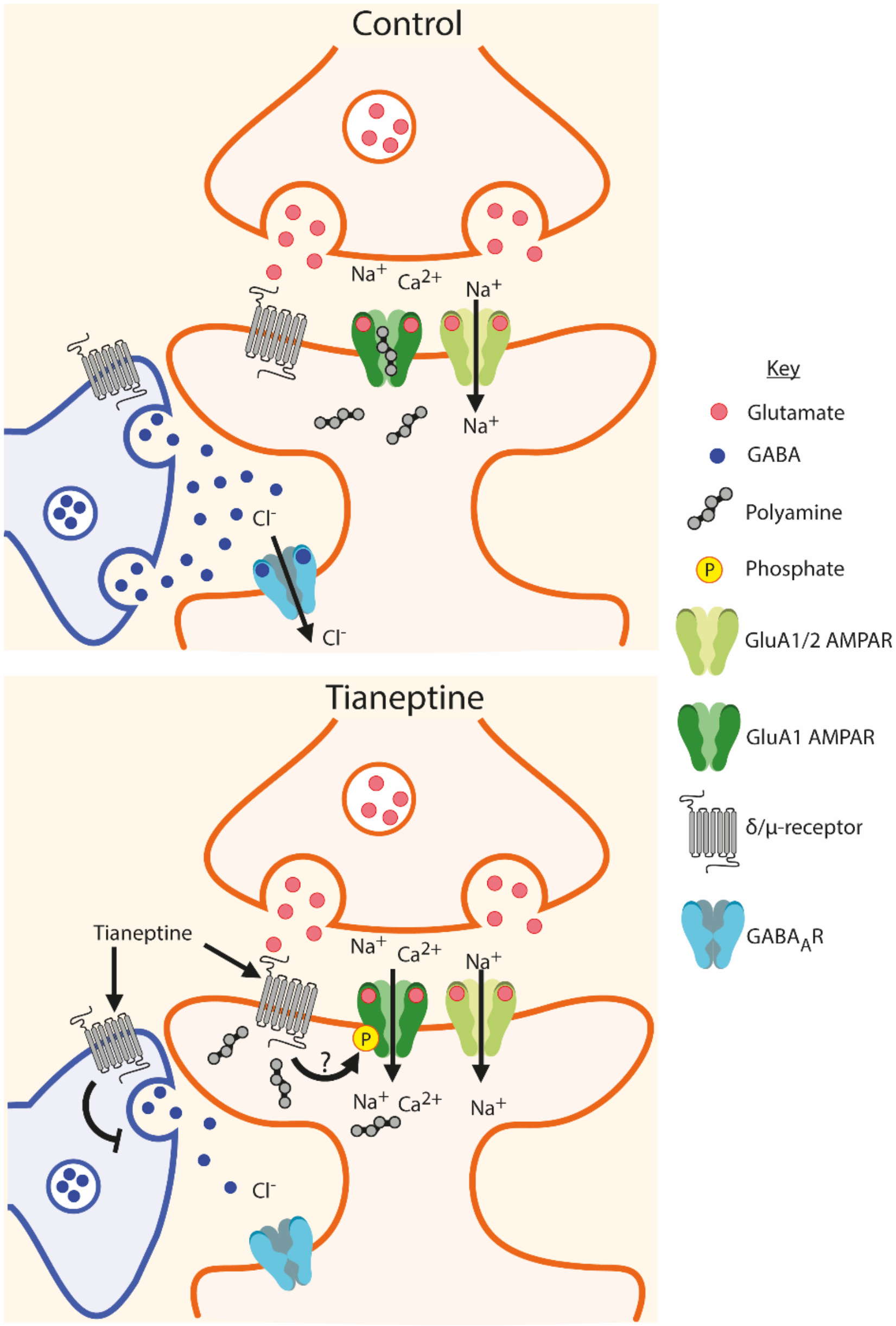

The influence of tianeptine on hippocampal excitatory and inhibitory synaptic transmission. Activation by tianeptine of μ- and δ-receptors expressed on postsynaptic CA1 pyramidal neurons (orange neuron) impairs the resident polyamine block of GluA1-AMPARs. This indirect facilitation glutamatergic transmission *via* opioid receptors may require phosphorylation of the GluA1 subunit, a biochemical modification known to influence the antidepressant-like actions of tianeptine in mice [5]. Additionally, activation by DAMGO of presynaptic μ-receptors located upon CA1 hippocampal GABA-ergic interneurons (blue neuron) decreases neurally evoked GABA release onto pyramidal cell neurons (disinhibition) [65]. Similarly, we found tianeptine (50 μM) to reduce the neurally-evoked GABA release onto mouse CA1 pyramidal neurons [66]. Note in mice the genetic deletion of μ-receptors of hippocampal somatostatin positive interneurons impairs the antidepressant-like effects of tianeptine in mice [6].
